# Severe acute respiratory syndrome coronavirus 2 vaccine breakthrough infections: A single metro-based testing network experience

**DOI:** 10.3389/fmed.2022.1031083

**Published:** 2022-11-25

**Authors:** Samantha S. Strickler, Annette Esper, Leona Wells, Anna Wood, Jennifer K. Frediani, Eric Nehl, Jesse J. Waggoner, Paulina A. Rebolledo, Joshua M. Levy, Janet Figueroa, Thanuja Ramachandra, Wilbur Lam, Gregory S. Martin

**Affiliations:** ^1^School of Medicine, Emory University, Atlanta, GA, United States; ^2^Nell Hodgson Woodruff School of Nursing, Emory University, Atlanta, GA, United States; ^3^Rollins School of Public Health, Atlanta, GA, United States; ^4^Hubert Department of Global Health, Rollins School of Public Health, Atlanta, GA, United States; ^5^Georgia Institute of Technology, Atlanta, GA, United States

**Keywords:** SARS-CoV-2, breakthrough infections, pandemic, partial vaccinations, COVID-19 vaccine

## Abstract

**Objectives:**

Understanding the incidence and characteristics that influence severe acute respiratory syndrome coronavirus 2 (SARS-CoV-2) vaccine breakthrough infections (VBIs) is imperative for developing public health policies to mitigate the coronavirus disease of 2019 (COVID-19) pandemic. We examined these factors and post-vaccination mitigation practices in individuals partially and fully vaccinated against SARS-CoV-2.

**Materials and methods:**

Adults >18 years old were voluntarily enrolled from a single metro-based SARS-CoV-2 testing network from January to July 2021. Participants were categorized as asymptomatic or symptomatic, and as unvaccinated, partially vaccinated, or fully vaccinated. All participants had confirmed SARS-CoV-2 infection based on standard of care (SOC) testing with nasopharyngeal swabs. Variant analysis by rRT-PCR was performed in a subset of time-matched vaccinated and unvaccinated individuals. A subgroup of partially and fully vaccinated individuals with a positive SARS-CoV-2 rRT-PCR was contacted to assess disease severity and post-vaccination mitigation practices.

**Results:**

Participants (*n* = 1,317) voluntarily underwent testing for SARS-CoV-2 during the enrollment period. A total of 29.5% of the population received at least one SARS-CoV-2 vaccine (*n* = 389), 12.8% partially vaccinated (*n* = 169); 16.1% fully vaccinated (*n* = 213). A total of 21.3% of partially vaccinated individuals tested positive (*n* = 36) and 9.4% of fully vaccinated individuals tested positive (*n* = 20) for SARS-CoV-2. Pfizer/BioNTech mRNA-1273 was the predominant vaccine received (1st dose = 66.8%, 2nd dose = 67.9%). Chronic liver disease and immunosuppression were more prevalent in the vaccinated (partially/fully) group compared to the unvaccinated group (*p* = 0.003, *p* = 0.021, respectively). There were more asymptomatic individuals in the vaccinated group compared to the unvaccinated group [*n* = 6 (10.7%), *n* = 16 (4.1%), *p* = 0.045]. C_T_ values were lower for the unvaccinated group (median 24.3, IQR 19.1–30.5) compared to the vaccinated group (29.4, 22.0–33.7, *p* = 0.004). In the vaccinated group (*n* = 56), 18 participants were successfully contacted, 7 were lost to follow-up, and 2 were deceased. A total of 50% (*n* = 9) required hospitalization due to COVID-19 illness. Adherence to nationally endorsed mitigation strategies varied post-vaccination.

**Conclusion:**

The incidence of SARS-CoV-2 infection at this center was 21.3% in the partially vaccinated group and 9.4% in the fully vaccinated group. Chronic liver disease and immunosuppression were more prevalent in the vaccinated SARS-CoV-2 positive group, suggesting that these may be risk factors for VBIs. Partially and fully vaccinated individuals had a higher incidence of asymptomatic SARS-CoV-2 and higher C_T_ values compared to unvaccinated SARS-CoV-2 positive individuals.

## Introduction

The underlying factors contributing to vaccine breakthrough infections (VBIs) following vaccination against severe acute respiratory syndrome coronavirus 2 (SARS-CoV-2) have yet to be fully elucidated. Initial data reported in phase III clinical trials examining SARS-CoV-2 vaccination demonstrated high efficacy, with reductions in the incidence and severity of coronavirus disease of 2019 (COVID-19) illness (1–3). Understanding the characteristics of SARS-CoV-2 VBIs, as well as identifying potential risk factors for their occurrence, is imperative for developing public health policies to mitigate the COVID-19 pandemic.

Preliminary reports describing SARS-CoV-2 VBIs have focused on at-risk populations, including skilled nursing facility residents and healthcare workers (4–7). VBI rates in these populations, prior to the emergence of the SARS-CoV-2 delta and omicron variants, were reported to range from 0.4 to 2.6%. Data demontrated that the clinical presentation of SARS-CoV-2 VBIs was less severe and that 24–64.6% of affected patients were asymptomatic (4, 5, 7, 8). This is consistent with laboratory findings of lower mean viral loads, as estimated by higher cycle threshold (C_T_) values compared to unvaccinated SARS-CoV-2 (+) patients (9).

To better characterize SARS-CoV-2 VBIs, we examined the incidence of SARS-CoV-2 in partially and fully vaccinated adults (asymptomatic and symptomatic) who obtained SARS-CoV-2 testing through a single metro-based testing network. Secondary objectives included examining clinical and virologic characteristics of infections following vaccination (partial and full), self-reported evaluation of disease severity, and individual post-vaccination mitigation practices.

## Materials and methods

### Population

Study participants were enrolled from several hospital-based and outpatient SARS-CoV-2 testing centers between January 4 and July 22, 2021 in the metro-Atlanta area (10, 11). These testing centers are open to the public and were employee testing sites for two hospital systems. Participants were enrolled if they were 18 years of age or older, seeking a standard of care (SOC) SARS-CoV-2 test or who had one collected within the previous 24 h of study enrollment, and had at least one dose of any SARS-CoV-2 vaccine with an FDA Emergency Use Authorization (EUA). Symptoms were assessed on the day of SARS-CoV-2 testing. Exclusion criteria included the inability to tolerate nasal swabs or to provide informed consent.

A partial vaccine breakthrough infection (pVBI) was defined as any SARS-CoV-2 infection after the first dose of any of the available three SARS-CoV-2 vaccines (Pfizer, Moderna, Janssen), but <14 days after the second dose. VBIs that occurred among fully vaccinated individuals were defined per CDC guidelines as those occurring ≥14 days post second dose of either the Pfizer or Moderna vaccines or single dose of the Janssen vaccine (5).

Individuals who agreed to be contacted for future research were contacted *via* phone after diagnosis and asked to complete the VBI questionnaire that included questions on disease severity and mitigation practices (Supplementary Material 1). A minimum of three attempts by phone was made to contact individuals, followed by an attempt *via* email. All follow-up calls were conducted from May to July 2021. Clinical and demographic variables were collected in a web-based database (REDCap, Nashville, TN, USA) (11).

### Vaccine availability

Severe acute respiratory syndrome coronavirus 2 vaccines were first available under EUA for the Phase 1 eligible population as defined by the Georgia Interim COVID-19 vaccination plan beginning December 2021 (12). On March 25, 2021, all individuals older than 16 years of age became eligible for vaccination in the state of Georgia. Pfizer/BioNTech SARS-CoV-2 vaccine became available in mid-December 2020. Moderna vaccine was available at the end of December 2020, and the Janssen vaccine became available the beginning of March 2021.

### Survey of mitigation practices

During concept development of this study, the CDC recommended the following mitigation guidelines for all individuals despite vaccination status: (1) Wearing a mask in crowded outdoor events, (2) Wearing a mask in indoor public settings, (3) Wearing a mask on public transportation with limited occupancy, (4) Wearing a mask during indoor gatherings with unvaccinated and vaccinated people from multiple households, and (5) Wearing a mask indoors with unvaccinated people who are at risk of severe infection or live with another person at risk (13). On May 13th, 2021, the CDC updated their mitigation guidelines and recommended that individuals fully vaccinated no longer needed to wear a mask or physically distance in any setting, indoors or outdoors unless required by another organization or workplace (e.g., healthcare settings). This recommendation occurred during the VBI questionnaire follow-up timeframe (14).

### Sample collection and analysis

Nasal swabs for rRT-PCR (real-time reverse transcription polymerase chain reaction) testing were collected by trained study personnel using flocked tapered swabs (PurFlock Ultra^®^ Dry Transport System, Puritan Medical Products, Guliford, ME, USA). Swabs were placed in a sterile tube containing 1 ml saline and stored at either 4°C for up to 72 h or −80°C until batch testing was completed. C_T_ values from SOC SARS-CoV-2 rRT-PCR were abstracted by chart review.

For variant rRT-PCR testing, a case-control study was developed whereby vaccinated SARS-CoV-2 cases were matched 1:2 with unvaccinated SARS-CoV-2 cases based on the date of symptom onset (± 2 weeks). A total of 500 μL of sample from cases and controls were extracted on an eMAG instrument (bioMeriéux Inc., Durham, NC, USA) and eluted in 50 μL of buffer. Eluates were immediately tested with a protocol including 3 rRT-PCRs: (1) an internally controlled SARS-CoV-2 assay (CDC N2 target and RNase P) an assay to detect specific spike single nucleotide mutations associated with variants of concern, and (2) a triplex assay to detect spike Δ69–70 and ORF1a Δ3675–3677 (15–17).

### Statistical analysis

Descriptive statistics for the study were reported as medians and interquartile ranges for continuous variables and counts with percentages for categorical variables. Shapiro–Wilk tests were used to check normality of continuous data. Two-group comparisons were conducted using student’s *t*-tests for normally distributed continuous data. Otherwise, Wilcoxon rank-sum tests were used. Categorical data was compared using chi-squared tests or Fisher’s exact tests for expected cell counts <5. Statistical significance was assessed at the 0.05 level. All statistical analysis was conducted using SAS 9.4 (Cary, NC, USA) and R Core Team (18).

## Results

### Patient population

**FIGURE 1:**
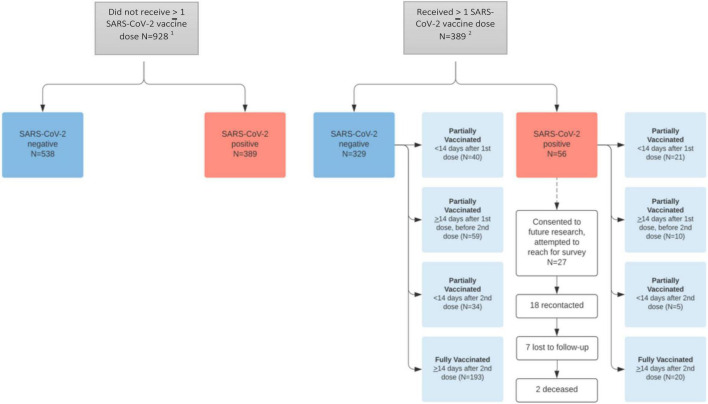
Study population. ^1^One undetermined result in “Did not receive >1 SARS-CoV-2 vaccine dose.” ^2^One undetermined result in “Received >1 SARS-CoV-2 vaccine dose” and three missing vaccination dates (and thus vaccine status) in “received >1 SARS-CoV-2 vaccine dose >SARS-CoV-2 negative.” Abbreviation: SARS-CoV-2, severe acute respiratory syndrome coronavirus 2.

Participants (*n* = 1,317) voluntarily underwent testing for SARS-CoV-2 during the enrollment period (Figure 1). At least one dose of a SARS-CoV-2 vaccine under EUA was received in 389 participants (29.5%), 169 were partially vaccinated (12.8%), and 213 were fully vaccinated (16.1%). In the partially vaccinated group, 61 participants were <14 days post 1st vaccine dose, 69 participants were >14 days post 1st vaccine dose, but had not received 2nd dose, and 39 participants were <14 days post 2nd vaccination (Supplementary Table 1). Three participants in the partially vaccinated group had missing dates of vaccination, and were excluded from analysis.

Among participants who received at least one SARS-CoV-2 vaccine dose, 329 had a negative test result, 56 had a positive result, and one had an indeterminate result. Of the 56 who tested positive, 36 (21.3%) were partially vaccinated and 20 (9.4%) were fully vaccinated. In the partially vaccinated SARS-CoV-2 (+) group, 21 were <14 days post 1st vaccination (58.3%) (Supplementary Table 1).

The Pfizer/BioNTech mRNA-1273 was the most prevalent vaccine administered (1st dose = 66.8%, 2nd dose = 67.9%, Supplementary Table 1), which corresponded with local availability. Among those partially vaccinated, 61.1% received the Pfizer/BioNTech mRNA-1273 vaccine (*n* = 22) and among those fully vaccinated, 85.0% received the Pfizer/BioNTech mRNA-1273 vaccine (*n* = 17). All participants who received Pfizer-BioNTech or Moderna vaccines received the same type of vaccine for both doses (Table 1).

**TABLE 1:** Demographics of SARS CoV-2 (+) unvaccinated and vaccinated [partially (*n* = 36) and fully (*n* = 20)] Groups.

Category	Total (*N* = 445)	Unvaccinated (*N* = 389)	Vaccinated (*N* = 56)	*P*-value
Age, median (IQR)	49 (32–60)	50.0 (33.0–60.0)	45.5 (28–61)	0.616
Sex, *n* (%)				0.730
Female	240 (53.9%)	211 (54.2%)	29 (51.8%)	
Male	205 (46.1%)	178 (45.8%)	27 (48.2%)	
Race, *n* (%)				0.364
White	114 (25.7%)	98 (25.2%)	16 (29.1%)	
Black/African American	280 (63.1%)	249 (64.0%)	31 (56.4%)	
Asian	22 (5.0%)	17 (4.4%)	5 (9.1%)	
Other	28 (6.3%)	25 (6.4%)	3 (5.5%)	
Ethnicity, *n* (%)				0.757
Non-Hispanic	417 (94.1%)	364 (93.8%)	53 (96.4%)	
Hispanic	26 (5.9%)	24 (6.2%)	2 (3.6%)	
Vaccine status, *n* (%)				–
One dose: <14 days ago	21 (37.5%)	0 (0.00%)	21 (37.5%)	
One dose: ≥14 days ago	10 (17.9%)	0 (0.00%)	10 (17.9%)	
Two doses: <14 days ago	5 (8.9%)	0 (0.00%)	5 (8.9%)	
Two doses: ≥14 days ago	20 (35.7%)	0 (0.00%)	20 (35.7%)	
First dose vaccine type, *n* (%)				–
Pfizer	39 (76.5%)	0 (0.00%)	39 (76.5%)	
Moderna	10 (19.6%)	0 (0.00%)	10 (19.6%)	
Johnson and Johnson	1 (2.0%)	0 (0.00%)	1 (2.0%)	
Not sure	1 (2.0%)	0 (0.00%)	1 (2.0%)	
Second dose vaccine type, *n* (%)				–
Pfizer	22 (88.0%)	0 (0.00%)	22 (88.0%)	
Moderna	3 (12.0%)	0 (0.00%)	3 (12.0%)	
Days since only first dose, median (IQR)	10 (5–19)	–	10.0 (5.0–19.0)	
Days since second dose, median (IQR)	54 (14, 88)	–	54.0 (14.0, 88.0)	

SARS-CoV-2, severe acute respiratory syndrome coronavirus 2; IQR, interquartile range.

### Participant characteristics

There were no demographic differences between the SARS-CoV-2 (+) unvaccinated (*n* = 389) and vaccinated (partially/fully, *n* = 56) groups (Table 1). In the vaccinated SARS-CoV-2 (+) group, the median age was 45.5 years (28–61 IQR), 51.8% were female, 56.4% were Black/African American, and 3.6% were Hispanic.

The most common previous medical conditions were hypertension (39%), diabetes (22%), and obesity (10%) with no difference between SARS CoV-2 (+) unvaccinated and vaccinated groups (Table 2). The vaccinated SARS-CoV-2 (+) group contained more participants with chronic liver disease (*p* = 0.003) and immunosuppression (transplant, chemotherapy, medications, or HIV) (*p* = 0.021) compared to the unvaccinated group. Additional data available describing medical conditions by vaccination dose and timeframe are available in the supplement (Supplementary Table 2).

**TABLE 2:** Medical conditions in SARS CoV-2 (+) unvaccinated and vaccinated [partially (*n* = 36) and fully (*n* = 20)] groups.

Category	Total (*N* = 445)	Unvaccinated (*N* = 389)	Vaccinated, (*N* = 56)	*P*-value
Number of conditions, median (IQR)	1 (0, 2)	1 (0, 2)	1 (0, 2)	0.231
Conditions				
High blood pressure	175 (39.3%)	154 (39.6%)	21 (37.5%)	0.765
Diabetes	96 (21.6%)	84 (21.6%)	12 (21.4%)	0.978
Obesity	45 (10.1%)	41 (10.5%)	4 (7.1%)	0.431
Chronic heart disease	29 (6.5%)	23 (5.9%)	6 (10.7%)	0.239
Chronic lung disease	13 (2.9%)	10 (2.6%)	3 (5.4%)	0.217
Chronic kidney disease	25 (5.6%)	19 (4.9%)	6 (10.7%)	0.111
Chronic liver disease	6 (1.3%)	2 (0.5%)	4 (7.1%)	**0.003**
Hemoglobin diseases	6 (1.3%)	5 (1.3%)	1 (1.8%)	0.556
Cancer	16 (3.6%)	14 (3.6%)	2 (3.6%)	1.000
Immunosuppression^1^	24 (5.4%)	17 (4.4%)	7 (12.5%)	**0.021**
Asthma	45 (10.1%)	38 (9.8%)	7 (12.5%)	0.526
Allergies	50 (11.2%)	45 (11.6%)	5 (8.9%)	0.559
Chronic sinus disease	9 (2.0%)	8 (2.1%)	1 (1.8%)	1.000
Other medical condition(s)	53 (11.9%)	45 (11.6%)	8 (14.3%)	0.557
No medical condition(s)	158 (35.5%)	140 (36.0%)	18 (32.1%)	0.574

^1^Immunosuppression included: From transplant, chemotherapy, medications, or HIV. SARS-CoV-2, severe acute respiratory syndrome coronavirus 2; IQR, interquartile range. Bold values are statistically significant.

In the vaccinated SARS-CoV-2 (+) group, 89.0% were symptomatic, compared to 95.5% in the unvaccinated group (*p* = 0.045; Table 3). There were no significant differences between the frequencies of most reported symptoms between the SARS-CoV-2 (+) unvaccinated and vaccinated groups. The most reported symptoms in the vaccinated SARS-CoV-2 (+) group were cough (61%), fatigue (52%), congestion (50%), and headache (50%). Chills were more common in the unvaccinated SARS-CoV-2 (+) group (*p* = 0.025). Additional data is available describing clinical presentation in all vaccinated individuals by vaccination dose and timeframe in the supplement (Supplementary Table 3).

**TABLE 3:** Clinical presentation of symptoms of SARS CoV-2 (+) unvaccinated and vaccinated [partially (*n* = 36) and fully (*n* = 20)] groups.

Category	Total (*N* = 445)	Unvaccinated (*N* = 389)	Vaccinated, (*N* = 56)	*P*-value
Symptom status, *n* (%)				
Asymptomatic	22 (4.9%)	16 (4.1%)	6 (10.7%)	**0.045**
Symptomatic	423 (95.1%)	373 (95.9%)	50 (89.3%)	
Days since symptom onset, median (IQR)	4 (2, 7)	4 (2, 7)	4 (2, 7)	0.985
Sum of symptoms, median (IQR)	5 (2–8)	5 (3–8)	4.5 (2–7)	0.369
Symptoms, *n* (%)				
Fever	191 (42.9%)	171 (44.0%)	20 (35.7%)	0.244
Chills	205 (46.1%)	187 (48.1%)	18 (32.1%)	**0.025**
Congestion	204 (45.8%)	176 (45.2%)	28 (50.0%)	0.504
Cough	298 (67.0%)	264 (67.9%)	34 (60.7%)	0.287
Headache	244 (54.8%)	216 (55.5%)	28 (50.0%)	0.437
Sore throat	142 (31.9%)	124 (31.9%)	18 (32.1%)	0.968
Fatigue	232 (52.1%)	203 (52.2%)	29 (51.8%)	0.955
Arthralgias	105 (23.6%)	91 (23.4%)	14 (25.0%)	0.791
Myalgias	141 (31.7%)	124 (31.9%)	17 (30.4%)	0.819
Photophobia	19 (4.3%)	16 (4.1%)	3 (5.4%)	0.720
Vomiting	62 (13.9%)	52 (13.4%)	10 (17.9%)	0.364
Nausea	95 (21.3%)	83 (21.3%)	12 (21.4%)	0.987
Diarrhea	111 (24.9%)	100 (25.7%)	11 (19.6%)	0.327
Abdominal pain	71 (16.0%)	62 (15.9%)	9 (16.1%)	0.980
Loss of taste or smell	134 (30.1%)	122 (31.4%)	12 (21.4%)	0.130
Shortness of breath	170 (38.2%)	149 (38.3%)	21 (37.5%)	0.908
Other symptom(s)	40 (9.0%)	33 (8.5%)	7 (12.5%)	0.326
No symptom(s)	22 (4.9%)	16 (4.1%)	6 (10.7%)	**0.045**

SARS-CoV-2, severe acute respiratory syndrome coronavirus 2; IQR, interquartile range. Bold values are statistically significant.

Participants were asked about post-vaccination mitigation practices. As shown in Supplementary Figure 3, more than 83% always continued to wear masks indoors when in contact with high-risk individuals, but mask wearing was less common in other indoor situations, and 61% always continued to wear masks in crowded outdoor situations.

### Virologic findings

C_T_ values in the SOC rRT-PCR were on average lower for unvaccinated SARS-CoV-2 (+) individuals (median 24.3, IQR 19.1–30.5) compared to vaccinated (partially/fully) SARS-CoV-2 (+) individuals (29.4, 22.0–33.7; Supplementary Figure 1). This difference was largely driven by the difference in values among symptomatic SARS-CoV-2 (+) individuals (Supplementary Figures 2A,B). C_T_ values were also significantly lower among symptomatic cases, regardless of vaccination status, versus asymptomatic individuals (Supplementary Figures 2C,D). Notably, C_T_ values did not differ based on vaccine type (Supplementary Figures 2E,F) or whether individuals were fully or partially vaccinated (Supplementary Figures 2C,G).

In a case-control study to test for mutations associated with SARS-CoV-2 variants, no differences in variant detection or distribution were observed between unvaccinated controls and partially or fully vaccinated individuals (Supplementary Table 4). rRT-PCR findings were consistent with Alpha variant (N501Y, Δ69/70, Δ3675–3677) being predominant in samples collected before May 2021 and with Delta variant (L452R, T478K) being predominant in samples collected in July.

### Disease severity and clinical outcomes

Of the 56 vaccinated SARS-CoV-2 (+) participants, 27 consented to participate in future research, and 18 participants were successfully contacted after diagnosis. Of the 9 that were unable to be contacted, 7 were lost to follow-up, and 2 were deceased (cause of death unable to be determined). Among the 18 participants, 8 were hospitalized, and 1 was admitted to the intensive care unit and required intubation (Supplementary Table 5). Participants spent on average 5.5 days in the hospital and 63% received some type of oxygen therapy (*n* = 5).

## Discussion

In this study, we found a relatively high incidence of VBIs, exceeding 20% in partially vaccinated individuals and nearly 10% in fully vaccinated individuals. There were no differences in basic demographics between the SARS-CoV-2 (+) unvaccinated and vaccinated (partially/fully) groups. Our study revealed a higher percentage of chronic liver disease and immunosuppression in vaccinated (partially/fully) SARS-CoV-2 (+) individuals compared to unvaccinated SARS-CoV-2 (+) individuals. The finding of immunosuppression is consistent with data showing increased risk of VBI in immunocompromised individuals (i.e., undergoing anti-neoplastic treatments) (19–21). To our knowledge, chronic liver disease has yet to be reported as a risk factor for VBIs. This finding may be attributable to cirrhosis-associated autoimmune dysfunction and an impaired immune response to vaccination, which has been previously reported with other vaccines (pneumococcus and hepatitis B) (22). More studies are needed to understand the impact of underlying medical conditions on VBIs.

When comparing clinical presentation of unvaccinated SARS-CoV-2 (+) to vaccinated SARS-CoV-2 (+) individuals, there were significantly more asymptomatic individuals in the vaccinated group (10.7 vs. 4.1%, *p* = 0.045). The incidence of asymptomatic VBIs has yet to be precisely defined but has been reported to range from 27–64% (4, 5). A handful of studies examining the incidence of asymptomatic SARS-CoV-2 infections in vaccinated versus unvaccinated populations have suggested a lower incidence of asymptomatic SARS-CoV-2 infections in vaccinated individuals compared to unvaccinated individuals (23–25). Our study contradicts this finding, which may be due to detection bias. It may be hypothesized that asymptomatic unvaccinated SARS-CoV-2 (+) individuals would be unlikely to seek testing, and therefore would have been underrepresented in this study. Another plausible explanation may be that asymptomatic individuals were pre-symptomatic at time of testing, and then later developed symptoms.

In our cohort, C_T_ values were higher in vaccinated SARS-CoV-2 (+) individuals compared to unvaccinated SARS-CoV-2 (+) individuals. C_T_ values were also significantly lower among symptomatic cases compared to asymptomatic individuals, regardless of vaccination status. To date there have been inconsistent reports, correlating C_T_ values with vaccination status. In a CDC report of outbreak infections in Massachusetts, there was no significant difference in C_T_ values between breakthrough cases and unvaccinated individuals (26). Additional studies, however, have reported higher C_T_ values in VBIs (9, 24, 27). In one study, C_T_ values were reported to be significantly higher as early as 12 days after vaccination (9). Previous studies examining the clinical time course of SARS-CoV-2 in unvaccinated individuals have demonstrated progressively rising CT values in the acutely symptomatic phase, followed by decreasing C_T_ values during convalesce (28, 29). As noted above, our study captured one clinical time point and did not examine the clinical time course of individual infections. Consequently, the C_T_ values reported have inherent variability as data was collected during different time points in the clinical course of SARS-CoV-2 infections. VBIs and C_T_ values requires further investigation with a focus on defining clinically significant disease and infectivity (30).

A case-control study of SARS-CoV-2 mutations nested into our overall study did not demonstrate a difference in the occurrence of any variant or the distribution of variants between vaccinated and unvaccinated cases. Rather, variants detected among vaccinated individuals were similar to those that were identified among unvaccinated individuals and within the community at the time of collection.

To our knowledge, this is the first study to examine SARS-CoV-2 post-vaccination mitigation strategies. Among participants that were contacted about mitigation practices after vaccination, the majority stated that they continued to wear masks indoors when around high-risk individuals and outdoors in crowded situations. Although our questionnaire was answered in May/June 2021 after the CDC’s recommendation for vaccinated people to discontinue wearing masks indoors, approximately 61% continued to wear masks indoors. It is unclear from our study what the association is between mitigation strategies and the development of VBIs.

Our study has several limitations. First, the overall number of vaccinated SARS-CoV-2 breakthrough cases was small; however, the data adds to our current knowledge on this subject and remains informative. Second, the incidence of breakthrough infections in this study may be influenced by selection bias. Individuals in this study willingly sought testing, were not specifically seeking medical treatment, and were agreeable to participating in research. Those with risk factors for severe SARS-CoV-2 (i.e., age >65, underlying medical conditions) may have also self-selected for vaccination and testing. Furthermore, there may also be inaccuracies in self-reported symptoms.

A fourth limitation was our inability to obtain mitigation strategy data on all partially or fully vaccinated SARS-CoV-2 (+) individuals. More data on adherence with various strategies would be useful to better inform public health policies. Understanding determinants of transmission and vaccine effectiveness is critical for developing prevention strategies. Lastly, most of the participants in our study received the Pfizer vaccine, which correlated with local availability. These data do not inform if vaccine product impacts VBI.

As demonstrated in our study population, unvaccinated individuals were more likely to test positive for SARS-CoV-2 and have symptomatic infections compared to vaccinated individuals, emphasizing the effectiveness of the vaccine. Our study, in addition to other published data, emphasizes how critical SARS-CoV-2 vaccines are for controlling the pandemic. With the ongoing emergence of variants of concern, understanding VBIs and the impact of mitigation strategies is critical for developing public health policies to address the COVID-19 pandemic.

## Conclusion

Despite controversial discussions surrounding SARS-CoV-2 vaccination, it has been repeatedly shown that they are highly efficacious in reducing COVID-19 infections, hospitalizations, and death, and this overwhelming supersedes the burden of VBIs. As has been universally reported, our study found that VBIs are infrequent and immunocompromised individuals are at increased risk of VBIs. Our study suggests that chronic liver disease may be an underlying co-morbidity for VBIs.

As VBIs are further investigated, future consideration should be given to establishing a universally accepted VBI definition reflective of the differences between asymptomatic VBIs, symptomatic VBIs, and ineffective immune responses to the vaccine. Furthermore, as vaccination rates increase and the number of partially vaccinated individuals increases, additional research will be needed to determine the efficacy and clinical burden of partial vaccination. There are a multitude of reasons why an individual only receives one dose of a multi-dose vaccine. Some reasons include but are not limited to side effects, scheduling time off, politics, or simply hesitancy. Further exploration of these factors will assist in the development of public health policies to mitigate the COVID-19 pandemic.

## Data availability statement

The raw data supporting the conclusions of this article will be made available by the authors, without undue reservation.

## Ethics statement

Ethical review and approval was not required for the study on human participants in accordance with the local legislation and institutional requirements. Written informed consent to participate in this study was provided by the participants’ legal guardian/next of kin.

## Author contributions

SS, AE, LW, JKF, PR, JW, EN, AW, JF GM, and WL contributed to the conception and design of the study. SS, AE, LW, JKF, PR, JW, EN, JL, AW, JF, TR, and GM were involved in drafting and revising of the manuscript. AW and JF performed the data analysis. SS, AE, LW, JKF, PR, JW, JL, JF, AW, TR, and GM were involved in interpretation of data. SS and AE agreed to take accountability for all aspects of the work if questions should arise. SS took responsibility for the manuscript as a whole. All authors contributed to the article and approved the submitted version.

## Supplementary material

Supplementary Figure 1 Cycle threshold (C_T_) values for unvaccinated SARS CoV-2 (+) individuals and vaccinated (partially and fully) SARS CoV-2 (+) individuals. Unvaccinated = 389, Vaccinated = 56 [partially (*n* = 36) and fully (*n* = 20)]. Abbreviation: SARS-CoV-2, severe acute respiratory syndrome coronavirus 2; CT, cycle threshold.

Supplementary Figure 2 Distribution of SARS-CoV-2 CT values **(A)** asymptomatic unvaccinated SARS-CoV-2 (+) (*n* = 16) and asymptomatic vaccinated SARS-CoV-2 (+) (*n* = 6), **(B)** symptomatic unvaccinated SARS-CoV-2 (+) (*n* = 373) and symptomatic vaccinated SARS-CoV-2 (+) (*n* = 50), **(C)** symptom status among vaccinated SARS-CoV-2 (+), **(D)** partially and fully vaccinated SARS-CoV-2 (+) **(E)** vaccine type for first dose, **(F)** vaccine type for second dose, **(G)** time post-vaccine dose. Abbreviation: SARS-CoV-2, severe acute respiratory syndrome coronavirus 2; CT, cycle threshold.

Supplementary Figure 3 Post-vaccination mitigation strategies in SARS CoV-2 (+) vaccinated subgroup (27 eligible, 18 successfully contacted). Abbreviation: SARS-CoV-2, severe acute respiratory syndrome coronavirus 2.

Supplementary Table 1 Demographics by vaccination dose and timeframe. *3 received Johnson and Johnson >14 days prior to test date so vaccine status is considered “Two doses: >14 days ago.” Abbreviations: SARS-CoV-2, severe acute respiratory syndrome coronavirus 2; IQR, interquartile range.

Supplementary Table 2 Medical conditions by vaccination dose and timeframe. ^1^Immunosuppression included: From transplant, chemotherapy, medications, or HIV. Abbreviations: SARS-CoV-2, severe acute respiratory syndrome coronavirus 2; IQR, interquartile range.

Supplementary Table 3 Clinical presentation by vaccination dose and timeframe. 3 received Johnson and Johnson >14 days prior to test date so vaccine status is considered “Two doses: >14 days ago.” Abbreviations: SARS-CoV-2, severe acute respiratory syndrome coronavirus 2; IQR, interquartile range.

Supplementary Table 4 Occurrence and distribution of specific mutations associated with SARS-CoV. 2 variants in vaccinated cases and unvaccinated controls. Abbreviations: SARS-CoV-2, severe acute respiratory syndrome coronavirus 2; IQR, interquartile range; rRT-PCR, real time reverse transcriptase polymerase chain reaction; CT, cycle threshold.

Supplementary Table 5 Breakthrough infections clinical information. Abbreviation: COVID-19, coronavirus disease 19.

Supplementary Material 1 Vaccine Breakthrough infection questionnaire. Abbreviation: COVID-19 = coronavirus disease 19.
