# Supplementary figures and images for: Severe acute respiratory syndrome coronavirus 2 vaccine breakthrough infections: A single metro-based testing network experience
Source: Front Med (Lausanne). 2022 Nov 25;9:1031083. doi: 10.3389/fmed.2022.1031083 (PMC9732086; doi:10.3389/fmed.2022.1031083)

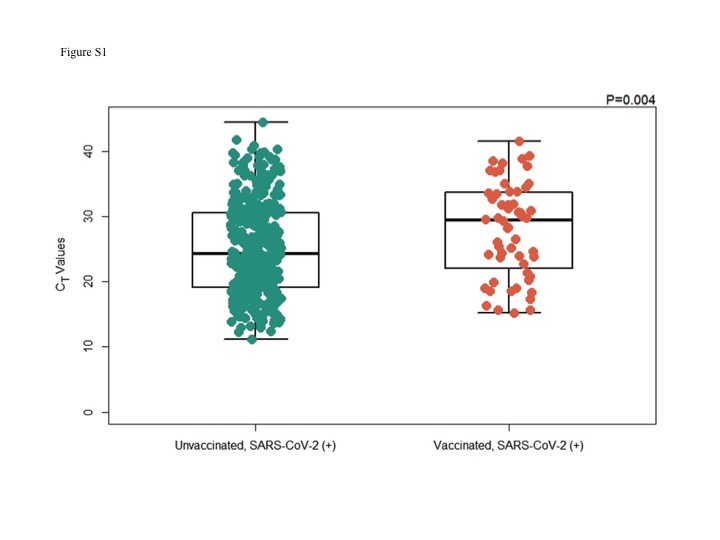

Supplement: Supplementary Figure 1 — Cycle threshold (CT) values for unvaccinated SARS CoV-2 (+) individuals and vaccinated (partially and fully) SARS CoV-2 (+) individuals. Unvaccinated = 389, Vaccinated = 56 [partially (n = 36) and fully (n = 20)]. Abbreviation: SARS-CoV-2, severe acute respiratory syndrome coronavirus 2; CT, cycle threshold. [file Image_1.JPEG]

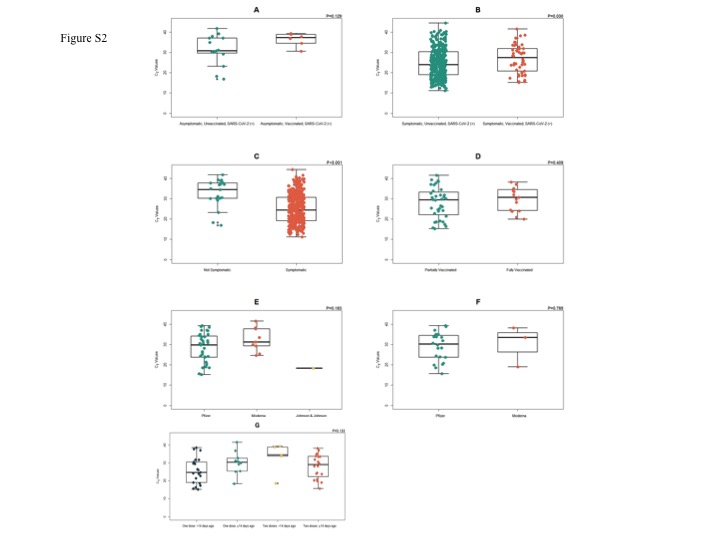

Supplement: Supplementary Figure 2 — Distribution of SARS-CoV-2 CT values (A) asymptomatic unvaccinated SARS-CoV-2 (+) (n = 16) and asymptomatic vaccinated SARS-CoV-2 (+) (n = 6), (B) symptomatic unvaccinated SARS-CoV-2 (+) (n = 373) and symptomatic vaccinated SARS-CoV-2 (+) (n = 50), (C) symptom status among vaccinated SARS-CoV-2 (+), (D) partially and fully vaccinated SARS-CoV-2 (+) (E) vaccine type for first dose, (F) vaccine type for second dose, (G) time post-vaccine dose. Abbreviation: SARS-CoV-2, severe acute respiratory syndrome coronavirus 2; CT, cycle threshold. [file Image_2.JPEG]

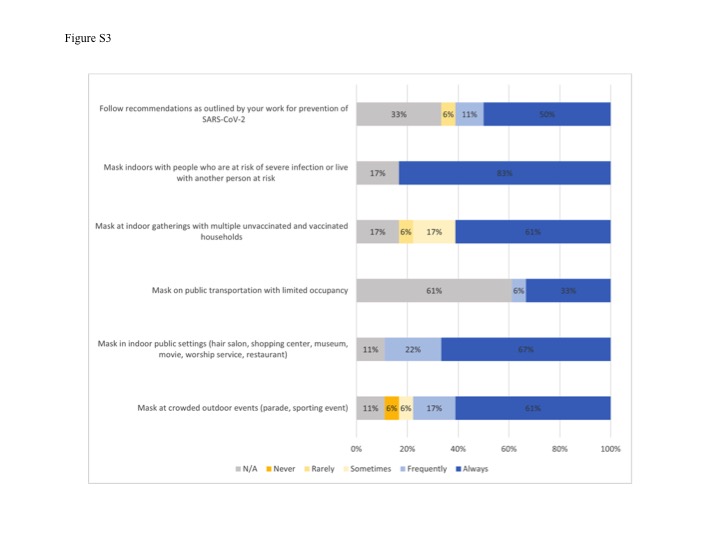

Supplement: Supplementary Figure 3 — Post-vaccination mitigation strategies in SARS CoV-2 (+) vaccinated subgroup (27 eligible, 18 successfully contacted). Abbreviation: SARS-CoV-2, severe acute respiratory syndrome coronavirus 2. [file Image_3.JPEG]
