# Supplementary material for: Severe acute respiratory syndrome coronavirus 2 vaccine breakthrough infections: A single metro-based testing network experience
Source: Front Med (Lausanne). 2022 Nov 25;9:1031083. doi: 10.3389/fmed.2022.1031083 (PMC9732086; doi:10.3389/fmed.2022.1031083)
Supplement: Supplementary Table 1 — Demographics by vaccination dose and timeframe. *3 received Johnson and Johnson >14 days prior to test date so vaccine status is considered “Two doses: >14 days ago.” Abbreviations: SARS-CoV-2, severe acute respiratory syndrome coronavirus 2; IQR, interquartile range. [file Data_Sheet_1.docx]

**Supplement 1.**

1. Were you hospitalized for COVID-19 disease? Yes/No.

If yes,

a. How many days were you in the hospital? ______ (number only)

b. Did you receive oxygen therapy of any kind? Yes/No

c. Were you admitted to the ICU? Yes/no. If yes, were you intubated at any time? Yes/No

2. After vaccination, how often do you wear your mask in crowded outdoor events (parade, sporting event)?

Almost Always Sometimes Every Once in a While Rarely Never

5 4 3 2 1

3. After vaccination, how often do you wear your mask in indoor public settings (i.e. hair salon, shopping center, museum, movie, worship service, restaurant)

Almost Always Sometimes Every Once in a While Rarely Never

5 4 3 2 1

4. After vaccination, how often do you wear your mask on public transportation with limited occupancy?

Almost Always Sometimes Every Once in a While Rarely Never

5 4 3 2 1

5. After vaccination, how often do you wear your mask during indoor gathering with unvaccinated and vaccinated people from multiple households?

Almost Always Sometimes Every Once in a While Rarely Never

5 4 3 2 1

6. After vaccination, how often do you wear your mask in indoors with unvaccinated people who are at risk of severe infection or live with another person at risk? Yes/No

Almost Always Sometimes Every Once in a While Rarely Never

5 4 3 2 1

7. After vaccination, how often do you follow recommendations as outlined by your work for prevention of COVID-19?

Almost Always Sometimes Every Once in a While Rarely Never

5 4 3 2 1

**Table S1**

| **Category** | **Overall**  **N=383** | **One dose:**  **<14 days ago**  **N=61** | **One dose:**  **>14 days ago**  **N=67** | **Two doses:**  **<14 days ago**  **N=39** | **Two doses:**  **>14 days ago**  **N=216** |
| --- | --- | --- | --- | --- | --- |
| Age, median (IQR) | 43 (29, 56) | 44.0 (27.0, 54.0) | 47.0 (34.0, 61.0) | 39.0 (27.0, 55.0) | 41.0 (29.0, 56.0) |
| Sex, n (%) |  |  |  |  |  |
| Female | 274 (71.5%) | 46 (75.4%) | 43 (64.2%) | 27 (69.2%) | 158 (73.1%) |
| Male | 109 (28.5%) | 15 (24.6%) | 24 (35.8%) | 12 (30.8%) | 58 (26.9%) |
| Race, n (%) |  |  |  |  |  |
| White | 195 (51.0%) | 22 (36.1%) | 34 (51.5%) | 23 (59.0%) | 116 (53.7%) |
| Black/African American | 141 (36.9%) | 30 (49.2%) | 25 (37.9%) | 12 (30.8%) | 74 (34.3%) |
| Asian | 28 (7.3%) | 6 (9.8%) | 6 (9.1%) | 2 (5.1%) | 14 (6.5%) |
| Other | 18 (4.7%) | 3 (4.9%) | 1 (1.5%) | 2 (5.1%) | 12 (5.6%) |
| Ethnicity, n (%) |  |  |  |  |  |
| Non-Hispanic | 361 (94.8%) | 59 (96.7%) | 65 (98.5%) | 37 (94.9%) | 200 (93.0%) |
| Hispanic | 20 (5.2%) | 2 (3.3%) | 1 (1.5%) | 2 (5.1%) | 15 (7.0%) |
| First dose vaccine type, n (%) |  |  |  |  |  |
| Pfizer | 219 (66.8%) | 35 (71.4%) | 35 (58.3%) | 19 (63.3%) | 130 (68.8%) |
| Moderna | 99 (30.2%) | 13 (26.5%) | 23 (38.3%) | 9 (30.0%) | 54 (28.6%) |
| Johnson & Johnson | 3 (0.9%) | 0 (0.00%) | 0 (0.00%) | 0 (0.00%) | 3 (1.6%) |
| Not sure | 7 (2.1%) | 1 (2.0%) | 2 (3.3%) | 2 (6.7%) | 2 (1.1%) |
| Second dose vaccine type, n (%) |  |  |  |  |  |
| Pfizer | 163 (67.9%) | 0 (0.00%) | 0 (0.00%) | 19 (63.3%) | 144 (68.6%) |
| Moderna | 73 (30.4%) | 0 (0.00%) | 0 (0.00%) | 9 (30.0%) | 64 (30.5%) |
| Not sure | 4 (1.7%) | 0 (0.00%) | 0 (0.00%) | 2 (6.7%) | 2 (1.0%) |
| Days since only first dose, median (IQR) | 14 (7, 21) | 6 (4.5, 10) | 21.0 (18.0, 26.0) | - | - |
| Days since second dose, median (IQR) | 44 (19, 82) | - | - | 8 (4, 11) | 53.0 (30.0, 92.0) |
| SARS-CoV-2 result |  |  |  |  |  |
| Negative | 326 (85.1%) | 40 (65.6%) | 57 (85.1%) | 34 (87.2%) | 195 (90.3%) |
| Positive | 56 (14.6%) | 21 (34.4%) | 9 (13.4%) | 5 (12.8%) | 21 (9.7%) |
| Undetermined | 1 (0.3%) | 0 (0.00%) | 1 (1.5%) | 0 (0.00%) | 0 (0.00%) |
| Ct values, median (IQR) | 28.0 (7.2) | 25.6 (7.3) | 31.5 (5.2) | 33.0 (8.4) | 27.7 (6.9) |

**Table S2**

| **Category** | **Overall**  **N=383** | **One dose:**  **<14 days ago**  **N=61** | **One dose:**  **>14 days ago**  **N=67** | **Two doses:**  **<14 days ago**  **N=39** | **Two doses:**  **>14 days ago**  **N=216** |
| --- | --- | --- | --- | --- | --- |
| Number of conditions, median (IQR) | 1 (0, 2) | 1 (0, 2) | 1 (0, 2) | 0 (0, 1) | 1 (0, 2) |
| Conditions |  |  |  |  |  |
| High blood pressure | 101 (26.4%) | 21 (34.4%) | 18 (26.9%) | 7 (17.9%) | 55 (25.5%) |
| Diabetes | 40 (10.4%) | 10 (16.4%) | 13 (19.4%) | 2 (5.1%) | 15 (6.9%) |
| Obesity | 27 (7.0%) | 2 (3.3%) | 5 (7.5%) | 2 (5.1%) | 18 (8.3%) |
| Chronic heart disease | 21 (5.5%) | 0 (0.00%) | 8 (11.9%) | 0 (0.00%) | 13 (6.0%) |
| Chronic lung disease | 11 (2.9%) | 0 (0.00%) | 1 (1.5%) | 0 (0.00%) | 10 (4.6%) |
| Chronic kidney disease | 13 (3.4%) | 2 (3.3%) | 4 (6.0%) | 0 (0.00%) | 7 (3.2%) |
| Chronic liver disease | 7 (1.8%) | 0 (0.00%) | 1 (1.5%) | 0 (0.00%) | 6 (2.8%) |
| Hemoglobin diseases | 1 (0.3%) | 1 (1.6%) | 0 (0.00%) | 0 (0.00%) | 0 (0.00%) |
| Cancer | 16 (4.2%) | 1 (1.6%) | 4 (6.0%) | 0 (0.00%) | 11 (5.1%) |
| Immunosuppression^1^ | 22 (5.7%) | 4 (6.6%) | 5 (7.5%) | 0 (0.00%) | 13 (6.0%) |
| Asthma | 56 (14.6%) | 14 (23.0%) | 13 (19.4%) | 2 (5.1%) | 27 (12.5%) |
| Allergies | 87 (22.7%) | 8 (13.1%) | 20 (29.9%) | 8 (20.5%) | 51 (23.6%) |
| Chronic sinus disease | 9 (2.3%) | 2 (3.3%) | 0 (0.00%) | 2 (5.1%) | 5 (2.3%) |
| Other medical condition(s) | 37 (9.7%) | 7 (11.5%) | 4 (6.0%) | 1 (2.6%) | 25 (11.6%) |
| No medical condition(s) | 146 (38.1%) | 20 (32.8%) | 23 (34.3%) | 21 (53.8%) | 82 (38.0%) |

**Table S3**

| **Category** | **Overall**  **N=383** | **One dose:**  **<14 days ago**  **N=61** | **One dose:**  **>14 days ago**  **N=67** | **Two doses:**  **<14 days ago**  **N=39** | **Two doses:**  **>14 days ago**  **N=216** |
| --- | --- | --- | --- | --- | --- |
| Symptom status, n (%) |  |  |  |  |  |
| Not symptomatic | 81 (21.1%) | 11 (18.0%) | 10 (14.9%) | 10 (25.6%) | 50 (23.1%) |
| Symptomatic | 302 (78.9%) | 50 (82.0%) | 57 (85.1%) | 29 (74.4%) | 166 (76.9%) |
| Days since symptom onset, median (IQR) | 3 (1, 5) | 3 (2, 5) | 3 (2, 6) | 4 (1, 5) | 3 (1, 4) |
| Sum of symptoms, median (IQR) | 3 (1, 6) | 3 (2, 6) | 3 (1, 5) | 3 (0, 6) | 3 (1, 6) |
| Symptoms, n (%) |  |  |  |  |  |
| Fever | 90 (23.5%) | 13 (21.3%) | 13 (19.4%) | 5 (12.8%) | 59 (27.3%) |
| Chills | 101 (26.4%) | 16 (26.2%) | 11 (16.4%) | 11 (28.2%) | 63 (29.2%) |
| Congestion | 168 (43.9%) | 25 (41.0%) | 28 (41.8%) | 18 (46.2%) | 97 (44.9%) |
| Cough | 156 (40.7%) | 33 (54.1%) | 27 (40.3%) | 10 (25.6%) | 86 (39.8%) |
| Headache | 172 (44.9%) | 32 (52.5%) | 29 (43.3%) | 17 (43.6%) | 94 (43.5%) |
| Sore throat | 140 (36.6%) | 17 (27.9%) | 22 (32.8%) | 15 (38.5%) | 86 (39.8%) |
| Fatigue | 161 (42.0%) | 29 (47.5%) | 26 (38.8%) | 19 (48.7%) | 87 (40.3%) |
| Arthralgias | 61 (15.9%) | 6 (9.8%) | 10 (14.9%) | 7 (17.9%) | 38 (17.6%) |
| Myalgias | 79 (20.6%) | 9 (14.8%) | 15 (22.4%) | 10 (25.6%) | 45 (20.8%) |
| Photophobia | 12 (3.1%) | 1 (1.6%) | 2 (3.0%) | 0 (0.00%) | 9 (4.2%) |
| Vomiting | 32 (8.4%) | 1 (1.6%) | 6 (9.0%) | 2 (5.1%) | 23 (10.6%) |
| Nausea | 75 (19.6%) | 13 (21.3%) | 10 (14.9%) | 8 (20.5%) | 44 (20.4%) |
| Diarrhea | 63 (16.4%) | 11 (18.0%) | 8 (11.9%) | 7 (17.9%) | 37 (17.1%) |
| Abdominal pain | 37 (9.7%) | 5 (8.2%) | 8 (11.9%) | 3 (7.7%) | 21 (9.7%) |
| Loss of taste or smell | 32 (8.4%) | 7 (11.5%) | 6 (9.0%) | 2 (5.1%) | 17 (7.9%) |
| Shortness of breath | 63 (16.4%) | 14 (23.0%) | 16 (23.9%) | 1 (2.6%) | 32 (14.8%) |
| Other symptom(s) | 20 (5.2%) | 4 (6.6%) | 3 (4.5%) | 2 (5.1%) | 11 (5.1%) |
| No symptom(s) | 81 (21.1%) | 11 (18.0%) | 10 (14.9%) | 10 (25.6%) | 50 (23.1%) |

**Table S4**

| **Category** | **Vaccinated Cases** | **Unvaccinated Controls** | **Potential Variant** |
| --- | --- | --- | --- |
| Patients, n | 25 | 50 |  |
| Repeat SARS-CoV-2 rRT-PCR positive, n (%) | 18 (72.0) | 35 (79.6) |  |
| C_T_ value, median (IQR) | 23.6 (20.2-27.1) | 26.2 (19.5-30.8) |  |
| Any mutation, n (% of positives) | 12 (66.7) | 22 (62.9) |  |
| *Genotype Detected, n (%)* |  |  |  |
| N501Y, Δ69/70, Δ3675-3677 | 6 (50.0) | 12 (54.5) | Alpha |
| L452R, T478K | 6 (50.0) | 5 (22.7) | Delta |
| L452R | 0 | 3 (13.6) | Epsilon |
| E484K, Δ3675-3677 | 0 | 1 (4.5) | Iota |
| N501Y | 0 | 1 (4.5) | ─ |

**Table S5: Breakthrough infections clinical information**

| **Category** | **Overall N=18** |
| --- | --- |
| Were you hospitalized for COVID-19 disease? |  |
| No | 10 |
| Yes | 8 |
| If you were hospitalized for COVID-19, how many days were you in the hospital? | 5.5 (4.0, 6.5) |
| If you were hospitalized for COVID-19, did you receive oxygen therapy of any kind? |  |
| No | 3 |
| Yes | 5 |
| If you were hospitalized for COVID-19, were you admitted to the ICU? |  |
| No | 6 |
| Yes | 1 |
| Unsure | 1 |
| If you were admitted to the ICU, how many days were you in the ICU? | N/A |
| If you were admitted to the ICU, were you intubated at any time? |  |
| Yes | 1 |

|  |  |
| --- | --- |
|  |  |
|  | |
